# Review on the Modeling of Electrostatic MEMS

**DOI:** 10.3390/s100606149

**Published:** 2010-06-21

**Authors:** Wan-Chun Chuang, Hsin-Li Lee, Pei-Zen Chang, Yuh-Chung Hu

**Affiliations:** 1 Institute of Applied Mechanics, National Taiwan University, Taipei, Taiwan; E-Mails: d96543004@ntu.edu.tw (W.-C.C.); changpz@mems.iam.ntu.edu.tw (P.-Z.C.); 2 MicroSystems Technology Center, Industrial Technology Research Institute, Taipei, Taiwan. E-Mail: SonyLee@itri.org.tw (H.-L.L.); 3 Department of Mechanical and Electromechanical Engineering, Center of Green Technology, National ILan University, ILan, Taiwan

**Keywords:** electrostatics, electromechanics, MEMS, pull-in voltage

## Abstract

Electrostatic-driven microelectromechanical systems devices, in most cases, consist of couplings of such energy domains as electromechanics, optical electricity, thermoelectricity, and electromagnetism. Their nonlinear working state makes their analysis complex and complicated. This article introduces the physical model of pull-in voltage, dynamic characteristic analysis, air damping effect, reliability, numerical modeling method, and application of electrostatic-driven MEMS devices.

## 1. Introduction

Micro-Electro-Mechanical Systems (MEMS) are an electromechanical integrated system where the feature size of components and the actuating range are within the micro-scale. Unlike traditional mechanical processing, manufacturing of MEMS device uses the semiconductor production process, which can be compatible with an integrated circuit, and includes surface micromachining and bulk micromachining. Due to the increasingly mature process technology, numerous sophisticated micro structural and functional modules are currently available. Therefore, greater optimized performance of the devices has been developed. Electrostatic-driven MEMS devices have advantages of rapid response, lower power consumption, and integrated circuit standard process compatibility. Among the present MEMS devices, many are electrostatic-driven MEMS devices, such as capacitive pressure sensors [1], comb drivers [2], micropumps [3], inkjet printer head [4], RF switches [5], and vacuum resonators [6].

Due to its simplicity of design and process, as well as convenience of integration with the integrated circuit processes to form a single-chip system, the electrostatic principle is commonly employed in sensing of MEMS or drive modules. However, due to the interaction between electrostatic force and structural behavior, namely the electromechanical coupling effects due to the coupling of multiple physical fields, such as stress fields and electrical fields, and since the system is nonlinear, instability of the pull-in often results, which leads to failures including stick, wear, dielectric changing, and breakdowns. Many studies have focused on common applications of electrostatic principle in MEMS devices, including: the instability when pull-in phenomenon occurs [7–37]; the deformation characteristic of microstructures subjected to electrostatic loads [18,38–41]; shape and position of drive electrodes [42–45]; dynamic response and optimization of electrostatic loads [46–57]; air damping effect [58–66], analysis method of chaos and bifurcation in electrostatic-driven systems [67,68], such as finite element method (FEM), finite difference method (FDM), and finite cloud meshless method (FCM) [68–73]; simulation software and systems of simulated dynamic behaviors, such as ANSYS, ABAQUS, COULOMB, MEMCAD, and macro models [69,72,74–78]; effects of routing parameters (voltage and temperature) on electrostatic force [79]; inherent nonlinear stiffness softening effect [70,80–82]; device reliability related failure modes and mechanisms; material selection; and reasonable design [8,32,38,83–92]. Without a thorough understanding of the effects of electrostatic force in MEMS systems, many practical phenomena, such as instability, nonlinearity and reliability, would have no scientific explanation. Thus, it is impossible to effectively explore and use the potential of MEMS technology. Under such circumstances, it is important and indispensable to study electro-mechanics of a micron scale structure under electrostatic loads.

**Figure 1.**
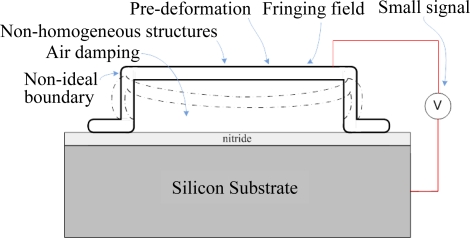
Nonlinear electromechanical coupling systems.

Accurate modeling the electrostatic microstructures is very challenging in virtue of the mechanical-electrical coupling effect and the nonlinearity of the structure and electrostatic force. Effects such as the non-ideal boundary conditions, fringing fields, pre-deformation due to the initial stresses, and non-homogeneous structures further complicate the modeling, as shown in Figure 1. A review paper [46] provided an overview of the fundamental research on nonlinear behaviors of electrostatic-driven microresonators, including direct and parametric resonances, parametric amplification, impacts, self-excited oscillations, and collective behaviors, such as localization and synchronization, which arise in coupled resonator arrays. Another review paper [56] presented an overview of the existing techniques before 2005 applied to the MEMS electrostatic actuation modeling and their dynamic behavior of the electromechanical system. A complete idealized model based on Euler-Bernoulli beam of an electrostatically actuated uniform beam is presented. Firstly, the energy expressions of the corresponding mechanical and electrical energy are derived. The kinetic energy and the bending and membrane strain energy are considered in the mechanical model. The fringing field is considered in the potential energy of electrostatic model. Two basic damping forces in MEMS, namely structural and viscous damping, are considered as well. The structural damping comes from the molecular interaction in the material due to deformation while the viscous damping comes from fluid that surrounds the moving microstructure. There are two types of viscous damping, namely couette flow damping and squeeze film camping. The governing equations are derived through substituting the above energy expressions into Lagrange equation. The static, transient, and oscillatory solutions of the model by numerical methods are illustrated respectively. A third review paper [57] presents an overview of the existing analytical models before 2007 for electrostatically actuated microdevices. General 3-dimensional nonlinear equations of motion for the coupled electromechanical fluid-structure interaction problem are outlined first. The microstructure is modeled as a solid elastic body. The air gap between the microstructure and fixed electrode is modeled as a homogeneous isotropic dielectric from the electric point of view while from the mechanical point of view, it is considered as a compressible Newtonian fluid. Deformations of microstructure are stated in Lagrange equation while the air gap and electrostatic field are stated in the Eulerian equations. The general 3-dimensional nonlinear model is too complicated to solve analytically. Therefore, simplified reduced order distributed models are illustrated along with such assumptions as beam and plate theories, squeeze film damping, and fringing field models.

The aforementioned three review articles provide complete idealized models based on the assumptions of ideal fixed boundaries, homogeneous structures, and without pre-deformations, while this work provides models considering the non-ideal boundary conditions, non-homogeneous structures, and pre-deformations due to initial stresses. Over the past few decades’ development, MEMS technologies are now capable of manufacturing many microsensing or actuating components employing standard Complementary-Metal-Oxide-Semiconductor (CMOS) processes, which are the so-called CMOS-MEMS. There is a gap between prototype and commercial product that must be filled out, namely the reliability testing of MEMS devices. This is exactly what the aforementioned three review articles lack. The main benefit of CMOS-MEMS is batch production using the well-developed standard CMOS facilities. However, apart from the electrical testing of circuits, the MEMS-side still requires the mechanical testing of microsensing or actuating components. The performance of microdevices depends on the constitutive properties of the thin-film structural materials of which they are made. It is known that thin-film properties can differ from bulk material ones. As a result, certain material properties are critical in device performance, which must be monitored in manufacturing to ensure the repeatability from device to device and wafer to wafer. However, the mechanical property extraction methods available in the literature for MEMS fabrication require additional measurement and actuating equipments or complicated test structure designs, which are not compatible with standard CMOS metrology technologies. To be compatible with CMOS metrology technologies, the best choice of test and pickup signals are both electrical. In the past decade, the mechanical property extraction for MEMS by electrostatic structures was developed. The following sections present the quasi-static pull-in physical model of MEMS devices, dynamic response analysis of microstructures, air damping effects, breakdown mechanism analysis of the components, numerical simulation, and the application on inline mechanical properties extraction of microstructures.

## 2. Development of Related Studies on Electro-Mechanics for MEMS Devices

### 2.1. Physical Model of Quasi-Static Pull-in Voltage

**Figure 2.**
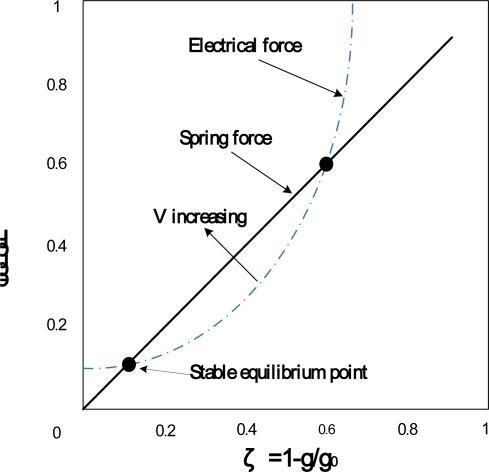
The electrical and spring force for voltage-controlled parallel-plate electrostatic actuator [93].

As shown in Figure 1, when there is an electrical potential difference between the beam and substrate, namely the drive voltage, the electrostatic attractive force will attract the beam downward while the elastic restoring force of the beam, namely the spring force, will restore the beam upward. In brief, the electrostatic attractive force and the elastic restoring force are kept in an equilibrium state. However, the deformation of the beam would cause a distribution change in its surface charges; thus, the electric field would redistribute and rework on the beam until a new equilibrium state is achieved. Therefore, Figure 1 is a nonlinear electromechanical coupling system. Figure 2 shows the variations of electrostatic force and spring force *versus* to the deformation of the beam.

**Figure 3.**
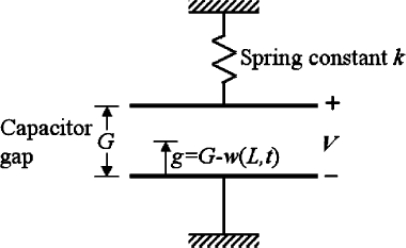
A discrete model of an equivalent spring and a parallel-plate capacitor [80].

The abscissa is the ratio of the beam deformation (g_0_-g) to the initial gap (g_0_) between beam and substrate while the ordinate is the forces. The spring force is proportional to the deformation of beam while the electrostatic force, namely the electrical force, is proportional to the square of the deformation. When the drive voltage is weak, the spring force can contend with the electrostatic force and keep the system in a stable equilibrium state. However, when the drive voltage achieves a critical value, the spring force can no longer contend with the electrostatic fore and throws the beam off balance. The critical value of drive voltage is referred to as pull-in voltage. The system is under an unstable equilibrium state at pull-in. The physical model of Figure 1 can be modeled by the analytical model shown in Figure 3 which consists of a parallel-plate capacitor suspended by a spring. The pull-in occurs when the deformation exceeds the one third of the initial gap of the parallel-plate. Thus, the key of analyzing electro-mechanics for MEMS devices is the study of the quasi-static pull-in properties.

**Figure 4.**
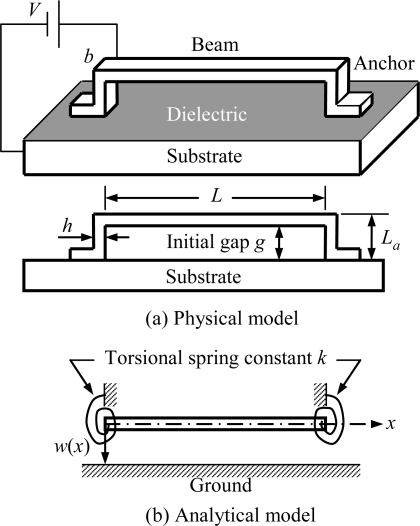
(a) Physical model, (b) Analytical model [34].

From 1994 to 1997 Senturia *et al.* published a series of research findings on electro-mechanics for MEMS devices. In 1994, Senturia [94] simulated a microbridge-shaped beam driven by electrostatic force as a lumped model of an equivalent spring and a parallel plate capacitor (Figure 3) in order to achieve the fundamental function forms of pull-in voltage, geometric dimension, and material parameters. Then in 1997, they proposed M-Test [8] technology, using semiconductor process technology to create three different micro test structures, namely, the microcantilever, the microbridge-shaped beam, and the microsector plate. They also produced micro- beams of different lengths to measure their pull-in voltage, respectively, in order to generalize the correcting factors based on those measured data and simulated numbers, and eventually modified the functional form based on the discrete model. In 2002, Pamidighantam *et al.* [19] simulated the system as a discrete system of an equivalent spring and a parallel plate capacitor. The stiffness of the equivalent spring and equivalent area of the parallel plate capacitor were obtained by CoventorWare, a commercial simulation software, in order to gain the pull-in voltage relation of the micro-bridge-shaped beams; however, the deviation was as high as 18%. In 2003, O’Mahony *et al.* [22] also analyzed the microbridge-shaped beams subjected to electrostatic loads using CoventorWare, and inferred the numerical solution of the microbridge-shaped beams by taking fringing capacitance effect, the efficiency of the plate-like phenomenon, and different boundaries into consideration. In 2005, Lishchynska *et al.* [95] derived the numerical solution of the pull-in voltage of cantilever beams, using the CoventorWare simulation software, and achieved an error within 4%. From 2004 to 2008, Krylov *et al.* published a series of research findings [7,12–14] on pull-in behavior of microstructures. In 2004, Krylov *et al.* [14] studied the transient nonlinear dynamics of microbeams subjected to electrostatic force, and developed a model based on the Galerkin procedure with normal modes which considered the effects about the distributed nonlinear electrostatic forces, nonlinear squeezed film damping, and rotational inertia of a mass carried by the beam. In 2006, Krylov *et al.* [13] developed simple expressions for electrostatic pressure with higher order corrections, mainly related to the curvature and slope of the electrode by using the perturbation theory. The results showed that tuning the ratio of the mechanical pressure, the string was with different pull-in behavior. In small initial pre-stress case, bistability of the string occurred. In 2008, Krylov *et al.* [7,12] reported on theoretical and experimental investigation of a multistability phenomenon in initially curved clamped-clamped microbeams subjected to a distributed electrostatic force. The results showed that the pull-in voltage of clamped-clamped with initially curved flexible was lower than a straight beam. From 2006 to 2009, Hu and co-workers proposed an approximate analytical model, taking into consideration such elements as initial stress, fringing capacitance effect, and elasticity boundaries [29,34,35,96,97]. For example, reference [34] presented an approximate analytical model to the pull-in voltage of a microbridge with elastic boundaries. The elasticity boundaries of the microbridge can be treated as a beam with torsional spring at both ends, and the conceptual diagram of a microbridge is shown in Figure 4.

**Figure 5.**
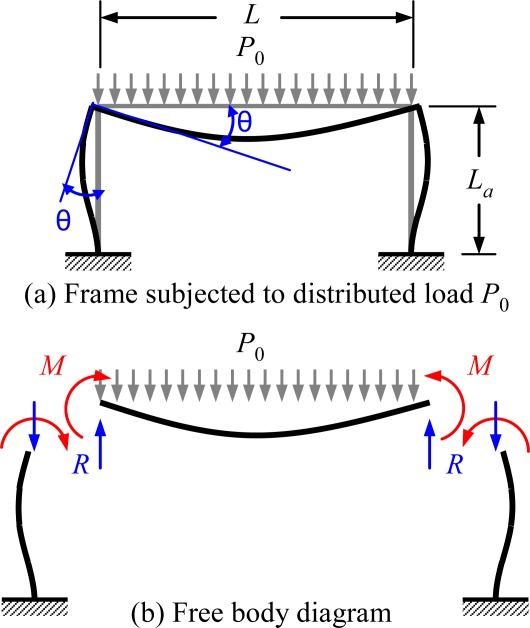
(a) Frame subjected to distributed load P_0_, (b) Free body diagram [34].

The beam is with length *L*, anchor height *L_a_*, width *b*, thickness *h*, and initial gap *g*, which subjected to a driving voltage *V*, resulted in a position-dependent deflection *w*(*x*). The equivalent torsional spring constant *k* can be expressed by the following equation [34]:

(1) $$k=\frac{M}{\theta }=\frac{4E\left({2I}_{a}^{2}L+I\cdot I_{a}L_{a}\right)}{{2I}_{a}L_{a}L+I_{a}L_{a}^{2}}$$

where *I* denoted the area moment of inertia, the subscript *a* denoted the anchor, and *E* denoted Young’s modulus. The reactive bending moment *M*, and the rotation angle *θ* were derived from a frame subjected to a uniform distributed load *P*_0_, as shown in Figure 5:

(2) $$M=\frac{P_{o}L^{2}}{12\left(1+0.5\frac{I}{I_{a}}\frac{L_{a}}{L}\right)}$$

(3) $$\theta =\frac{P_{o}L^{2}}{24E\left(2\frac{I_{a}}{L_{a}}+\frac{I}{L}\right)}$$

Based on the Euler’s beam model and minimum energy method, the pull-in voltage *V_PI_* of a micro-bridge was [34]:

(4) $$V_{\mathrm{PI}}^{2}=\frac{\sigma_{0}\int_{0}^{L}2\mathrm{bh}{\left(\phi^{\prime }\right)}^{2}\mathrm{dx}+E\int_{0}^{L}2I{\left(\phi^{\prime \prime }\right)}^{2}\mathrm{dx}+\left({8\mathrm{EI}}_{a}/L_{a}\right){\left(\phi^{\prime }\right)}_{x=L}^{2}}{\varepsilon \left(c_{1}+{2c}_{2}\eta_{\mathrm{PI}}+{3c}_{3}\eta_{\mathrm{PI}}^{2}\right)}$$

where *σ*_0_ denoted the initial stress, and *ϕ* was first natural mode of a beam having torsional spring at both ends given by [132]:

(5) $$\phi (x)=\left[\text{sin}\left(\frac{\beta x}{L}\right)-\text{sinh}\left(\frac{\beta x}{L}\right)\right]+\gamma \left[\text{cos}\left(\frac{\beta x}{L}\right)-\text{cosh}\left(\frac{\beta x}{L}\right)-\frac{{\beta L}_{a}}{2L}\text{sinh}\left(\frac{\beta x}{L}\right)\right]$$

(6) $$\gamma =\frac{\text{sinh}\;\beta -\text{sin}\;\beta }{\text{cos}\;\beta -\text{cosh}\;\beta -\frac{{\beta L}_{a}}{2L}\text{sinh}\;\beta }.$$

Besides, *β* should satisfie the following equation:

(7) $${\left(\frac{\mathrm{kL}}{\mathrm{EI}}\right)}^{2}+\frac{\mathrm{kL}}{\mathrm{EI}}\frac{2\beta \left(\text{sin}\;\beta \text{cosh}\;\beta -\text{cos}\;\beta \text{sinh}\;\beta \right)}{1-\text{cos}\;\beta \text{cosh}\;\beta }+\frac{{2\beta }^{2}\text{sin}\;\beta \text{sinh}\;\beta }{1-\text{cos}\;\beta \text{cosh}\;\beta }=0.$$

Equation (4) shows an obvious physical meaning. The first term shows that the pull-in voltage was dependent on initial residual stress, the second one was dependent on beam flexibility, and the third one was dependent on elastic boundary condition. The findings had greater physical significance than the previous studies we motioned above, which employed numerical methods in studies of electro-mechanics for MEMS Devices.

### 2.2. Microstructure Dynamic Response Analysis

**Figure 6.**
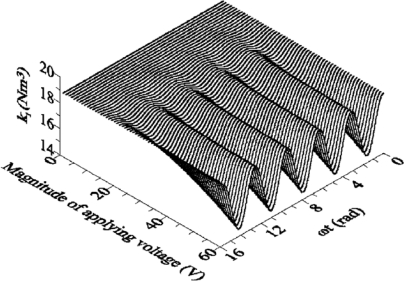
The first modal stiffness variation [80].

In the movement process, beams are affected by the interaction between electrostatic force, elasticity-restoring force, and damping force. As a result, the equation of motion in coupling is often a simultaneous partial differential equation of an electrostatic force equation, an Euler beam equation, and an air-damping equation, which explain the dynamic actions of the devices in all three spatial dimensions. It would be very difficult to solve this equation using only a numerical method. Therefore, mathematical operations, such as state-variable analysis and basic function expansion methods, are usually employed to translate a partial differential equation of infinite dimensions into a system of ordinary differential equations of finite dimensions. This is known as the reduced order method [75,98]. Younis *et al.* [75] presented a reduced-order model to analyze the behavior of microbeams actuated by electrostatic force. The model was obtained by discretizing the distributed-parameter system using Galerkin procedure into a finite-degree-of-freedom system, which considered the effects about moderately large deflections, dynamic loads, linear and nonlinear elastic restoring forces, the nonlinear electrostatic force generated by the capacitors, and the coupling between the mechanical and electrostatic force. However, under the reduced order method, it would be very difficult to analyze the dynamic actions of the devices when air damping is taken into consideration; while other related parameters are still obtained through the numerical method, thus, analysis efficiency has not been improved. To solve this problem, Clark [99] determined an effective method for analyzing the dynamic actions of MEMS devices, by which the complicated system is divided into several basic structural units, and then the equivalent circuit model, consisting of those basic structures, is built using the simulation protocols between similar systems. Wen [100] employed a model analysis method based on linear disposal near the bias point in order to analyze the AC small signal in frequency domain of beams. Zhang [101] set an electrostatic-driven cantilever beam equivalent to a single-degree-of-freedom model to conduct its analytical form, and used a feedback mechanism to realize the coupling. Time and frequency domain analyses were conducted. In the time domain analysis, stronger driving voltage leads to more obvious beams overshoot. In the frequency domain analysis, the natural frequency of the cantilever beams would gradually reduce with an increase of voltage. Hu [80] established an analysis model for dynamic characteristics and stability of electrostatic-driven devices, and found that the stiffness of a microstructure will be softened periodically with the frequency of applied voltage. The variation of stiffness increases with the magnitude of applied voltage (Figure 6).

**Figure 7.**
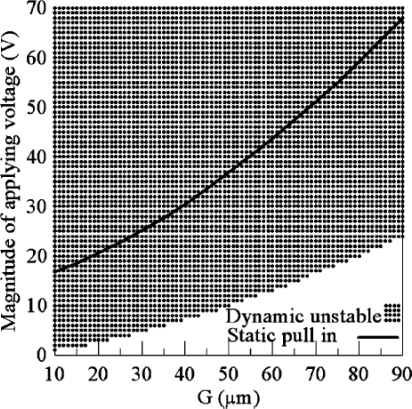
Comparison between dynamic instability and static pull-in [80].

The dynamical pull-in voltage may be lower than the static one (Figure 7).

**Figure 8.**
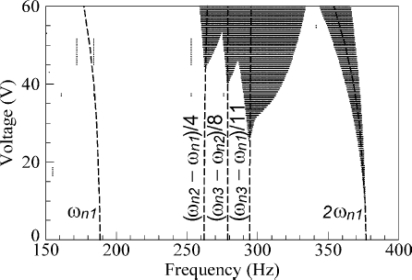
Dynamical instable region of a microcantilever subjected to an AC voltage [80].

Furthermore, the instable regions of the dynamical pull-in expand with the increasing of applied voltage, as shown in the dot-area of Figure 8. If the structure acts in small deformation condition, the beam can be considered as linear. On the contrary, for large deformations, non-linear structural effects have to be considered. For solving the non-linearity problem, one can use the finite element analysis (FEA) to obtain a non-linear cubic term. Moreover, it would be helpful to predict non-linear mechanical stiffness behavior [71,72,102,103]. The governing equation of a vibrating system with cubic stiffness non-linearity can be written as [50]:

(8) $$m\ddot{x}+k_{1}x+k_{3}x^{3}+c\dot{x}=f(\upsilon )$$

where *c*, *f*(*v*), *m*, *k*_1_, *k*_3_, and *x* represent the equivalent damping coefficient, the equivalent external periodical force depending on drive voltage *v*, the equivalent mass, the equivalent linear stiffness coefficient, the equivalent non-linear stiffness coefficient, and the deformation respectively.

### 2.3. Air Damping Effect

Microstructures, which move relatively along a vertical direction, are widely used in MEMS devices, such as microaccelerometers [104,105]. During movement, the structure would be affected by the air damping between device and substrates. When the devices are in greater working amplitude vibration, the air damping force would be obviously strengthened. Thus, it is critical to establish an air damping model of components under movement. Blech [63] analyzed seismic accelerometers, and treated them as a damping device consisting of two plates with squeeze film of gas between them. Besides, the squeeze film damping cutoff frequencies can be solved analytically from the lowest eigenvalue of the Helmholtz equation. Andrews [106] employed the theoretical model to predict the frequency response of isolated rectangular plates oscillating normal to each other and the prediction results agreed well with the experimental data. Darling [107] presented an analytical method based upon a Green’s function solution to linearize Reynolds equation, and allowed the forces from compressible squeeze film damping to be rapidly calculated for arbitrary acoustic venting conditions along the edges of a moveable structure. For laminar flows under relatively high pressure or relatively large space between the plates, Reynolds equations are used for analysis. The air flow can be treated as a continuum media. The degree of rarefaction depends on the so-called Knudsen number, Kn, defined as [108], where d is the gap distance and l the mean-free-path:

(9) $$k_{n}=\frac{\lambda }{d}$$

Veijola [59] utilized the circuit model to calculate the effective viscosity in a narrow gap between the moving surfaces. The damping coefficient was obtained by using Blech model [63], and the spring constant was estimated by curve fitting experimental measurements. Besides, he used an effective coefficient *η_eff_* of viscosity for accounting for the slip-flow condition, where *η*_0_ is the coefficient of viscosity under atmospheric pressure:

(10) $$\eta_{\mathrm{eff}}=\frac{\eta_{0}}{1+9.658K_{n}^{1.159}}$$

In 1999, Li *et al.* [109] studied the damping effects of beams in a resonance state. In 2001, Yang [110] analyzed the relation between the air damping effects of MEMS devices and their working efficiency. In 2005, Zhang[70] suggested a simplified electrostatic-driven cantilever beam model, which considers nonlinear air damping and could be used to study the relationship between resonance response parameters and nonlinear dynamics. In the same year, Wang [60], took squeezed gas effects into consideration, and established an air damping model of a microplate structure in order to obtain a characteristic change of a pressed film within a resonance pressed cycle of motion through the finite difference method. The study showed that squeezed air effects must be taken into consideration in a theoretical analysis model; otherwise, the air damping effects would be over measured. The increased plate dimension of microstructures would increase the damping force, and the increasing speed of damping force is greater than that of the size of the microstructures. In addition, the increased resonance frequency would increase the air damping effect. In 2007, Wang [111] further established a nonlinear dynamics model of a microresonator by taking slip boundaries and squeezed air effects into consideration. Also in 2007, Zhu [112] developed an analytical formula of air damping force and the damping coefficient of a microplate structure by converting the governing equation along a vertical direction into the Fourier series. The study showed that the air damping coefficient is inversely proportional to the third power of the thickness of the air gap between the two plates.

### 2.4. Numerical/CAD Methods

**Figure 9.**
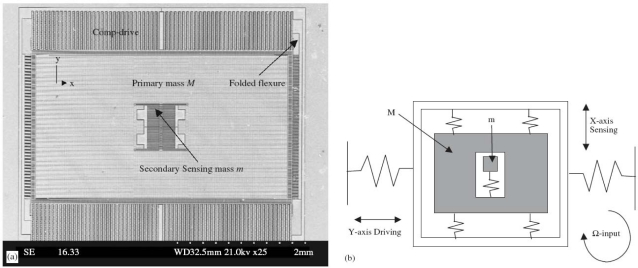
SEM of a gyroscope (a) and its lumped model (b) [50].

The development process of a MEMS system is complicated, involving product design, manufacturing, packing and systemic integration. Like an IC circuit and its common mechanical structures, MEMS devices can use computer aided design (CAD) to facilitate their performance, reliability, reduce the development cycle and costs. The main difference is that MEMS CAD is still a work in progress. For electronic products design, the technology of electronic design automation (EDA) serves as a platform to enable circuit designers to design and analyze, with the help of a computer and model libraries provided by foundries and related design kits, in order to complete the design, development, and testing of devices in the most economic and efficient method. SPICE, SABER, and Simulink are software commonly used. For mechanical products design, MDA (Mechanical Design Automation) has numerous and large-scale common software to aid design, manufacturing, and analysis, such as IDEAS, UGII, and ProPEngineer. A linking device between EDA and MDA is required for MEMS CAD to determine multiple physical coupling effects, which increases the development difficulties of MEMS CAD. Numerical simulation of MEMS devices mainly uses numerical methods, such as the finite element method, the boundary element method, and can simulate the actions of various structural components with high accuracy. Its drawbacks are high computation complexity and low analysis efficiency. Therefore, related studies have explored how to reduce the large amount of computations. Hung [113] examined the methods to define grids, which are able to generate the most effective reduced model during the process of devices’ working. Stewart [114] developed a set of simulation methods, which can be used for microstructures in small vibraction situation. Swart [115] invented a computer-aided software, named AutoMM, which can automatically produce dynamic models for microstructures. Commercial FEA/BEA tools usually used for the MEMS design, like ANSYS, ABAQUS, Maxwell, CoventorWare, CFDRC, IntelliCAD, CAEMEMS, SESES ,and SOLIDIS. For example, Figure 9 shows the SEM of a gyroscope and its lumped model.

**Figure 10.**
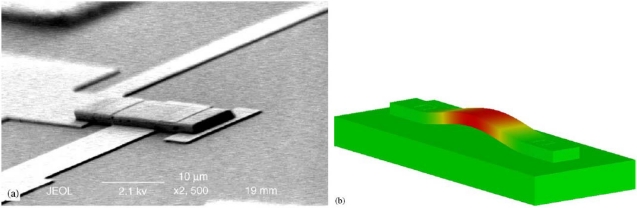
SEM of a microbeam resonator (a) and its 1st modal shape simulated by CoventorWare (b) [50].

The suspended MEMS devices can always be treated as linear lumped model under the assumption of small displacement. The whole structure can be considered as linear massless springs (flexures parts) connected to the rigid mass (the proof mass).Then, one can use the FEA tools to obtain the spring constants and the equivalent mass [118]. Besides, one can use these tools to solve the governing equations with given boundary conditions to know the mode shape of microstructures (Figure 10).

### 2.5. Breakdown Mechanism Analysis of Microstructures

**Figure 11.**
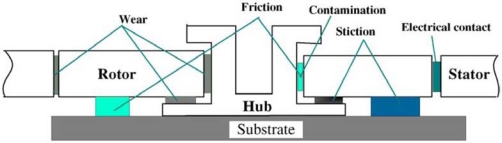
Tribology issues during micromotor operation [38].

The breakdown of MEMS devices means that the devices are unable to achieve their expected functions. Zhang [117] discussed major breakdown modes and breakdown mechanisms of various MEMS devices, such as microswitches, micromotors, and comb drivers [118–124]. Mariani [87–92] studied the breakdown mechanism of multi-scale structures subjected to drop impacts using a finite element method. Komvopoulos [125] proposed that the major breakdown modes of MEMS devices are friction, wear, and stick (Figure 11), which should be avoided to improve the reliability of the devices.

In 1992, Bart [126] proposed a dynamic model that involved analysis of kinetic friction, and found that the roughness of the surface has great impact on the friction force of the devices. Tai [127] established a model for friction movement to calculate the static friction coefficient of the devices, and suggested that due to the narrow gap between the rotor and the hub, contact wear is easily produced. Gabriel [122] found that the hub would be dramatically corroded or deformed at high speeds as the wear shortens the life span of the devices and limits their performance; however, a bracing structure could be adopted to avoid wear. Stick between the contact faces could prevent the devices from repeated operation or even working. Spengen [118] reported that the surface roughness of devices is a critical element that affects stick. Ren *et al.* [128] employed self-assembled monolayers (SAMs) to reduce the stick of devices surfaces.

## 3. Extracting the Mechanical Properties Utilizing Electromechanical Behavior of the Microstructures

Electrostatic-driven MEMS devices have been widely used in various sensing and actuating, and can be used in biosensors or to extract mechanical properties of thin film materials. In the past decade, all the important discoveries on the technology of calculated mechanical properties of thin film materials came from the research team of Senturia [8] at MIT. The key point is to generalize the threshold voltage of devices and the experimental formula of MEMS mechanical properties. The advantage of this technology is the simplicity of the measurement of the threshold voltage; while the disadvantage is that the experimental formula must be used in conjunction with certain test micro structures. If the devices changes, then the formula has to change as well. It is also difficult to use the experimental formula in non-ideal boundaries, such as with pre-deformation or non-homogenous sections. Different from Senturia, Hu [29,30,34,35,96,97,129–131] proposed a nonlinear mechanical and electrical coupling system of a micro structure for a pull-in voltage approximate analytical model, which involves non-ideal boundaries, fringing capacitance effect, and residual stresses. Hu also developed a fully electrical signal testing method for the measurement of the mechanical properties of thin film materials, which can be used in wafer-level tests to examine the Young’s modulus and residual stresses of micro structures. Reference [131] presented a formula about the relationship between Young’s modulus, residual stress and pull-in voltage of micro test beams which considered the effects about fringing field capacitance, the distributed characteristics of micro test beams, and the electromechanical coupling effect:

(11) $$\begin{array}{l} \sigma_{0}\left(\mathrm{bh}\int_{0}^{L}{\phi^{\prime }}^{2}\mathrm{dx}\right)+E\left(I\int_{0}^{L}{\phi^{\prime \prime }}^{2}\mathrm{dx}\right)\\ =\frac{{\varepsilon V}^{2}}{2}\int_{0}^{L}\left[\frac{{2b\phi }^{2}}{{\left(g-\eta \phi \right)}^{3}}+\frac{{0.33125b}^{0.25}\phi^{2}}{{\left(g-\eta \phi \right)}^{2.25}}+\frac{{0.795h}^{0.5}\phi^{2}}{{\left(g-\eta \phi \right)}^{2.5}}\right]\mathrm{dx} \end{array}$$

where *η_PI_* denoted the value of *η* at pull-in state which can be expressed as:

(12) $$\begin{array}{l} \eta_{\mathrm{PI}}\int_{0}^{L}\left[\frac{{2b\phi }^{2}}{{\left(g-\eta_{\mathrm{PI}}\phi \right)}^{3}}+\frac{{0.33125b}^{0.25}\phi^{2}}{{\left(g-\eta_{\mathrm{PI}}\phi \right)}^{2.25}}+\frac{{0.795h}^{0.5}\phi^{2}}{{\left(g-\eta_{\mathrm{PI}}\phi \right)}^{2.5}}\right]\mathrm{dx}\\ -\int_{0}^{L}\left[\frac{b\phi }{{\left(g-\eta_{\mathrm{PI}}\phi \right)}^{2}}+\frac{{0.265b}^{0.25}\phi }{{\left(g-\eta_{\mathrm{PI}}\phi \right)}^{1.25}}+\frac{{0.53h}^{0.5}\phi }{{\left(g-\eta_{\mathrm{PI}}\phi \right)}^{1.5}}\right]\mathrm{dx}=0 \end{array}$$

Equation (12) can be solved by numerical method. Substituting the value of *η_PI_* into equation (11), than we can obtain to the correlation between the pull-in voltage *V_PI_* and the structural material parameters σ_0_ and *E*:

(13) $${S\sigma }_{0}+\mathrm{BE}=V_{\mathrm{PI}}^{2}$$

where the parameters *S* and *B* depend on the geometrical parameters of micro test beam and are given as:

(14) $$S=\frac{\int_{0}^{L}{\mathrm{bh}\phi \prime }^{2}\mathrm{dx}}{\int_{0}^{L}\;\frac{\varepsilon }{2}\left[\frac{{2b\phi }^{2}}{{\left(g-\eta_{\mathrm{PI}}\phi \right)}^{3}}+\frac{{0.33125b}^{0.25}\;\phi^{2}}{{\left(g-\eta_{\mathrm{PI}}\phi \right)}^{2.25}}+\frac{{0.795h}^{0.5}\;\phi^{2}}{{\left(g-\eta_{\mathrm{PI}}\phi \right)}^{2.5}}\right]\mathrm{dx}}$$

(15) $$B=\frac{\int_{0}^{L}{I\phi \prime \prime }^{2}\mathrm{dx}}{\int_{0}^{L}\frac{\varepsilon }{2}\left[\frac{{2b\phi }^{2}}{{\left(g-\eta_{\mathrm{PI}}\phi \right)}^{3}}+\frac{{0.33125b}^{0.25}\phi^{2}}{{\left(g-\eta_{\mathrm{PI}}\phi \right)}^{2.25}}+\frac{{0.795h}^{0.5}\phi^{2}}{{\left(g-\eta_{\mathrm{PI}}\phi \right)}^{2.5}}\right]\mathrm{dx}}$$

where *b*, *E*, *h*, *I*, *L*, and *σ*_0_ represent the beam width, Young’s modulus, thickness, area inertia moment of beam cross section, beam length, and the initial stress, and *ϕ* was first natural mode of a fixed-fixed beam given by [132]. Therefore, one can extract Young’s modulus and residual stress easily by substituting the measured pull-in voltages of the two test beams with different length:

(16) $$\left\{\begin{array}{c} \sigma_{0}\\ E \end{array}\right\}={\left[\begin{array}{cc} S_{1} & B_{1}\\ S_{2} & B_{2} \end{array}\right]}^{-1}\left\{\begin{array}{c} V_{\mathrm{PI}1}^{2}\\ V_{\mathrm{PI}2}^{2} \end{array}\right\}$$

The testing technology is able to conduct inline measurements and monitoring of wafer fabrication, and uses existing semiconductor measurement equipment, as they are adequate for semiconductor and MEMS processes.

## 4. Conclusions

Analysis of the electro-mechanics of electrostatic-driven MEMS devices is complex due to the coupling of several energy domains. Besides, the electromechanical coupling effects will cause the pull-in instability, nonlinear response, reliability issues during the system operation. This article has reviewed related literature on electrostatic-driven MEMS devices, including a physical model of quasi-static pull-in voltage about how the physical quantities, like residual stress, elastic boundary, structural flexibility, fringing field capacitance to affect pull-in voltage, dynamic characteristic analysis about the dynamic behavior when system operates, air damping effects about the relation between air damping coefficient and geometry of structure, reliability about the failure mode and failure mechanisms of various devices, numerical modeling method about how to generate the most effective reduced model to fit the real system, and application. By the further understanding of the interaction mechanisms of these significant topics, it is helpful for developing the optimization techniques and applications in MEMS field.
